# Bis{μ-*N*′-[1-(5-bromo-2-oxidophen­yl)ethyl­idene]benzene­sulfono­hydrazidato}-κ^3^ *O* ^2^,*N*′:*N*;κ^3^ *N*:*O* ^2^,*N*′-bis­[(dimethyl sulfoxide-κ*O*)copper(II)]

**DOI:** 10.1107/S1600536808002201

**Published:** 2008-01-25

**Authors:** Hapipah M. Ali, Musalem Laila, Razali M. Rizal, Seik Weng Ng

**Affiliations:** a Department of Chemistry, University of Malaya, 50603 Kuala Lumpur, Malaysia

## Abstract

In the title centrosymmetric dinuclear complex, [Cu_2_(C_15_H_11_BrN_2_O_3_S)_2_(C_2_H_6_OS)_2_], the Cu^II^ ion is *N*,*O*-chelated by a dianionic ligand, monocoordinated by the sulfonamide N atom of a symmetry-related ligand and coordinated by an O atom from a dimethyl sulfoxide ligand, forming a distorted square-planar coordination geometry.

## Related literature

For the structure of 2′-[1-(2-hydroxy­phen­yl)ethyl­idene]benzene­sulfonohydrazide, see: Ali *et al.* (2007 ▶).
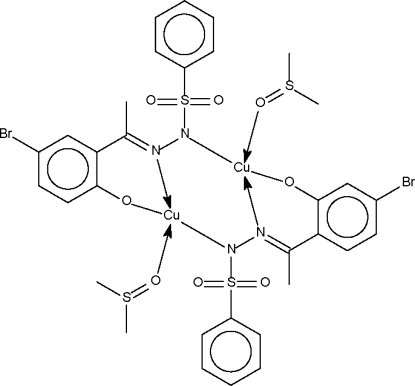


## Experimental

### 

#### Crystal data

[Cu_2_(C_15_H_11_BrN_2_O_3_S)_2_(C_2_H_6_OS)_2_]
*M* *_r_* = 1017.77
Triclinic, 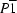

*a* = 8.0831 (1) Å
*b* = 10.4972 (2) Å
*c* = 12.9481 (2) Å
α = 68.157 (1)°
β = 74.928 (1)°
γ = 70.691 (1)°
*V* = 950.56 (3) Å^3^
*Z* = 1
Mo *K*α radiation
μ = 3.49 mm^−1^
*T* = 123 (2) K
0.40 × 0.31 × 0.20 mm

#### Data collection

Bruker APEXII diffractometer
Absorption correction: multi-scan (*SADABS*; Sheldrick, 1996 ▶) *T*_min_ = 0.335, *T*_max_ = 0.542 (expected range = 0.308–0.497)
12330 measured reflections
4318 independent reflections
3788 reflections with *I* > 2σ(*I*)
*R*_int_ = 0.027

#### Refinement

*R*[*F* ^2^ > 2σ(*F* ^2^)] = 0.039
*wR*(*F* ^2^) = 0.151
*S* = 1.21
4318 reflections
238 parameters
H-atom parameters constrained
Δρ_max_ = 1.75 e Å^−3^
Δρ_min_ = −0.89 e Å^−3^

### 

Data collection: *APEX2* (Bruker, 2005 ▶); cell refinement: *SAINT* (Bruker, 2005 ▶); data reduction: *SAINT*; program(s) used to solve structure: *SHELXS97* (Sheldrick, 2008 ▶); program(s) used to refine structure: *SHELXL97* (Sheldrick, 2008 ▶); molecular graphics: *X-SEED* (Barbour, 2001 ▶); software used to prepare material for publication: *publCIF* (Westrip, 2008 ▶).

## Supplementary Material

Crystal structure: contains datablocks global, I. DOI: 10.1107/S1600536808002201/lh2588sup1.cif

Structure factors: contains datablocks I. DOI: 10.1107/S1600536808002201/lh2588Isup2.hkl

Additional supplementary materials:  crystallographic information; 3D view; checkCIF report

## Figures and Tables

**Table 1:** Selected bond lengths (Å)

Cu1—O1	1.894 (3)
Cu1—O4	1.986 (3)
Cu1—N1	1.967 (3)
Cu1—N2^i^	2.026 (3)

Symmetry code: (i) 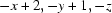.

**Fig. 1.**
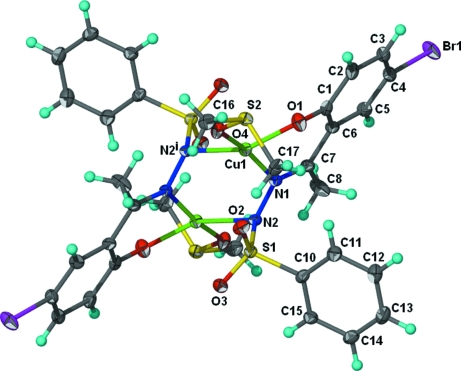
The molecular structure with displacement ellipsoids drawn at the 50% probability level, and H atoms shown as spheres of arbitrary radii [symmetry code: (i) -x + 2, -y + 1, -z].

## supplementary crystallographic information

### Experimental

The Schiff base ligand was synthesized by refluxing 5-bromo-2-hydroxyacetophenone (0.6 g, 2.8 mmol) with benzene sulfonohydrazide (0.48 g,2.8 mmol)in ethanol for 2 h. The ligand then was refluxed with Copper (II) acetate for 5 h. The brown crystal were obtained by recrystalization the product from DMSO.

### Refinement

All H atoms were placed in calculated positions (C–H = 0.95–0.98 Å) and were included in the refinement in the riding-model approximation with *U*_iso_(H) set to 1.2*U*_eq_(C) or 1.5*U*_eq_(C) for methyl H atoms.

### Crystal data

**Table:**

[Cu_2_(C_15_H_11_BrN_2_O_3_S)_2_(C_2_H_6_OS)_2_]	*Z* = 1
*M**_r_* = 1017.77	*F*(000) = 510
Triclinic, *P*1	*D*_x_ = 1.778 Mg m^−^^3^
Hall symbol: -P 1	Mo *K*α radiation, λ = 0.71073 Å
*a* = 8.0831 (1) Å	Cell parameters from 7546 reflections
*b* = 10.4972 (2) Å	θ = 2.7–31.0°
*c* = 12.9481 (2) Å	µ = 3.49 mm^−^^1^
α = 68.157 (1)°	*T* = 123 K
β = 74.928 (1)°	Block, green
γ = 70.691 (1)°	0.40 × 0.31 × 0.20 mm
*V* = 950.56 (3) Å^3^	

### Data collection

**Table:**

Bruker APEXII diffractometer	4318 independent reflections
Radiation source: medium-focus sealed tube	3788 reflections with *I* > 2σ(*I*)
Graphite	*R*_int_ = 0.027
φ and ω scans	θ_max_ = 27.5°, θ_min_ = 1.7°
Absorption correction: multi-scan (*SADABS*; Sheldrick, 1996)	*h* = −10→10
*T*_min_ = 0.335, *T*_max_ = 0.542	*k* = −13→13
12330 measured reflections	*l* = −16→16

### Refinement

**Table:**

Refinement on *F*^2^	Primary atom site location: structure-invariant direct methods
Least-squares matrix: full	Secondary atom site location: difference Fourier map
*R*[*F*^2^ > 2σ(*F*^2^)] = 0.039	Hydrogen site location: inferred from neighbouring sites
*wR*(*F*^2^) = 0.151	H-atom parameters constrained
*S* = 1.21	*w* = 1/[σ^2^(*F*_o_^2^) + (0.0827*P*)^2^ + 1.8365*P*] where *P* = (*F*_o_^2^ + 2*F*_c_^2^)/3
4318 reflections	(Δ/σ)_max_ = 0.001
238 parameters	Δρ_max_ = 1.75 e Å^−^^3^
0 restraints	Δρ_min_ = −0.89 e Å^−^^3^

### Fractional atomic coordinates and isotropic or equivalent isotropic displacement parameters (Å^2^)

**Table:**

	*x*	*y*	*z*	*U*_iso_*/*U*_eq_	
Br1	0.33316 (6)	0.23924 (5)	0.54963 (4)	0.03789 (17)	
Cu1	1.09563 (6)	0.32031 (5)	0.10740 (4)	0.02020 (15)	
S1	1.13219 (13)	0.63204 (10)	0.05283 (8)	0.0213 (2)	
S2	1.41379 (13)	0.06832 (10)	0.18255 (8)	0.0230 (2)	
O1	1.0528 (4)	0.2167 (3)	0.2622 (3)	0.0285 (6)	
O2	1.2628 (4)	0.4956 (3)	0.0677 (3)	0.0278 (6)	
O3	1.1615 (4)	0.7489 (3)	−0.0472 (3)	0.0292 (7)	
O4	1.3150 (4)	0.1704 (3)	0.0840 (2)	0.0253 (6)	
N1	0.9142 (4)	0.4879 (3)	0.1354 (3)	0.0195 (6)	
N2	0.9352 (4)	0.6205 (3)	0.0558 (3)	0.0198 (6)	
C1	0.8917 (5)	0.2298 (4)	0.3191 (3)	0.0219 (8)	
C2	0.8558 (6)	0.1098 (5)	0.4082 (3)	0.0269 (9)	
H2	0.9472	0.0236	0.4212	0.032*	
C3	0.6946 (6)	0.1120 (5)	0.4770 (3)	0.0274 (9)	
H3	0.6759	0.0294	0.5370	0.033*	
C4	0.5591 (6)	0.2368 (5)	0.4575 (3)	0.0249 (8)	
C5	0.5843 (5)	0.3557 (4)	0.3695 (4)	0.0243 (8)	
H5	0.4889	0.4393	0.3569	0.029*	
C6	0.7481 (5)	0.3565 (4)	0.2975 (3)	0.0208 (7)	
C7	0.7691 (5)	0.4889 (4)	0.2075 (3)	0.0217 (8)	
C8	0.6221 (7)	0.6230 (5)	0.1999 (5)	0.0392 (12)	
H8A	0.6631	0.7033	0.1428	0.059*	
H8B	0.5875	0.6396	0.2731	0.059*	
H8C	0.5198	0.6135	0.1788	0.059*	
C10	1.1190 (5)	0.6831 (4)	0.1713 (3)	0.0220 (8)	
C11	1.1381 (6)	0.5793 (5)	0.2752 (4)	0.0269 (8)	
H11	1.1558	0.4822	0.2833	0.032*	
C12	1.1307 (6)	0.6200 (5)	0.3673 (4)	0.0318 (9)	
H12	1.1418	0.5505	0.4393	0.038*	
C13	1.1072 (6)	0.7618 (5)	0.3547 (4)	0.0327 (10)	
H13	1.1064	0.7884	0.4174	0.039*	
C14	1.0849 (6)	0.8649 (5)	0.2507 (4)	0.0304 (9)	
H14	1.0656	0.9622	0.2429	0.036*	
C15	1.0910 (6)	0.8258 (4)	0.1583 (4)	0.0249 (8)	
H15	1.0761	0.8957	0.0869	0.030*	
C16	1.6285 (6)	0.0005 (5)	0.1132 (4)	0.0294 (9)	
H16A	1.6190	−0.0542	0.0690	0.044*	
H16B	1.7060	−0.0613	0.1694	0.044*	
H16C	1.6786	0.0797	0.0631	0.044*	
C17	1.4719 (6)	0.1775 (5)	0.2371 (4)	0.0310 (9)	
H17A	1.3669	0.2215	0.2823	0.046*	
H17B	1.5168	0.2519	0.1746	0.046*	
H17C	1.5640	0.1193	0.2844	0.046*	

### Atomic displacement parameters (Å^2^)

**Table:**

	*U*^11^	*U*^22^	*U*^33^	*U*^12^	*U*^13^	*U*^23^
Br1	0.0284 (3)	0.0359 (3)	0.0357 (3)	−0.01063 (19)	0.00829 (19)	−0.0030 (2)
Cu1	0.0171 (3)	0.0210 (3)	0.0222 (3)	−0.00342 (18)	−0.00075 (18)	−0.00929 (19)
S1	0.0190 (5)	0.0249 (5)	0.0240 (5)	−0.0098 (4)	0.0014 (3)	−0.0118 (4)
S2	0.0192 (5)	0.0238 (5)	0.0253 (5)	−0.0066 (4)	−0.0020 (4)	−0.0070 (4)
O1	0.0205 (14)	0.0309 (15)	0.0248 (14)	0.0000 (12)	−0.0005 (11)	−0.0063 (12)
O2	0.0179 (14)	0.0312 (16)	0.0391 (17)	−0.0064 (12)	0.0007 (12)	−0.0200 (13)
O3	0.0320 (17)	0.0352 (16)	0.0255 (15)	−0.0200 (14)	0.0015 (13)	−0.0095 (13)
O4	0.0220 (14)	0.0278 (14)	0.0245 (14)	0.0011 (11)	−0.0040 (11)	−0.0130 (12)
N1	0.0206 (16)	0.0187 (15)	0.0205 (15)	−0.0074 (12)	−0.0020 (12)	−0.0063 (12)
N2	0.0201 (16)	0.0205 (15)	0.0214 (15)	−0.0094 (12)	−0.0014 (12)	−0.0074 (12)
C1	0.0202 (18)	0.0271 (19)	0.0204 (18)	−0.0047 (15)	−0.0025 (14)	−0.0113 (15)
C2	0.030 (2)	0.0246 (19)	0.0213 (19)	0.0012 (16)	−0.0058 (16)	−0.0085 (15)
C3	0.036 (2)	0.0252 (19)	0.0199 (18)	−0.0089 (17)	−0.0040 (17)	−0.0047 (15)
C4	0.024 (2)	0.029 (2)	0.0213 (18)	−0.0088 (16)	0.0002 (15)	−0.0081 (16)
C5	0.0196 (18)	0.0247 (19)	0.028 (2)	−0.0055 (15)	−0.0014 (15)	−0.0090 (16)
C6	0.0190 (18)	0.0193 (17)	0.0245 (18)	−0.0061 (14)	−0.0008 (15)	−0.0081 (14)
C7	0.0190 (18)	0.0202 (18)	0.0255 (19)	−0.0070 (14)	0.0012 (15)	−0.0084 (15)
C8	0.030 (2)	0.021 (2)	0.046 (3)	−0.0012 (18)	0.011 (2)	−0.0033 (19)
C10	0.0177 (18)	0.0252 (19)	0.0275 (19)	−0.0056 (15)	−0.0009 (15)	−0.0149 (16)
C11	0.025 (2)	0.026 (2)	0.029 (2)	−0.0045 (16)	−0.0016 (16)	−0.0115 (16)
C12	0.029 (2)	0.038 (2)	0.027 (2)	−0.0038 (19)	−0.0042 (17)	−0.0122 (18)
C13	0.030 (2)	0.044 (3)	0.032 (2)	−0.0112 (19)	−0.0001 (18)	−0.023 (2)
C14	0.030 (2)	0.030 (2)	0.037 (2)	−0.0118 (18)	0.0038 (18)	−0.0194 (19)
C15	0.024 (2)	0.0241 (19)	0.027 (2)	−0.0099 (16)	0.0023 (16)	−0.0099 (16)
C16	0.023 (2)	0.027 (2)	0.038 (2)	−0.0021 (16)	−0.0020 (17)	−0.0153 (18)
C17	0.031 (2)	0.038 (2)	0.031 (2)	−0.0086 (19)	−0.0062 (18)	−0.0181 (19)

### Geometric parameters (Å, °)

**Table:**

Br1—C4	1.902 (4)	C5—H5	0.9500
Cu1—O1	1.894 (3)	C6—C7	1.472 (5)
Cu1—O4	1.986 (3)	C7—C8	1.501 (6)
Cu1—N1	1.967 (3)	C8—H8A	0.9800
Cu1—N2^i^	2.026 (3)	C8—H8B	0.9800
S1—O3	1.445 (3)	C8—H8C	0.9800
S1—O2	1.450 (3)	C10—C11	1.388 (6)
S1—N2	1.626 (3)	C10—C15	1.390 (6)
S1—C10	1.772 (4)	C11—C12	1.391 (6)
S2—O4	1.537 (3)	C11—H11	0.9500
S2—C17	1.779 (4)	C12—C13	1.387 (7)
S2—C16	1.781 (4)	C12—H12	0.9500
O1—C1	1.310 (5)	C13—C14	1.389 (7)
N1—C7	1.295 (5)	C13—H13	0.9500
N1—N2	1.423 (4)	C14—C15	1.388 (6)
N2—Cu1^i^	2.026 (3)	C14—H14	0.9500
C1—C2	1.410 (6)	C15—H15	0.9500
C1—C6	1.438 (5)	C16—H16A	0.9800
C2—C3	1.372 (6)	C16—H16B	0.9800
C2—H2	0.9500	C16—H16C	0.9800
C3—C4	1.388 (6)	C17—H17A	0.9800
C3—H3	0.9500	C17—H17B	0.9800
C4—C5	1.374 (6)	C17—H17C	0.9800
C5—C6	1.407 (6)		
			
O1—Cu1—N1	89.77 (13)	C1—C6—C7	122.5 (4)
O1—Cu1—O4	91.02 (13)	N1—C7—C6	119.4 (3)
N1—Cu1—O4	167.44 (13)	N1—C7—C8	120.8 (4)
O1—Cu1—N2^i^	153.28 (14)	C6—C7—C8	119.7 (4)
N1—Cu1—N2^i^	93.89 (13)	C7—C8—H8A	109.5
O4—Cu1—N2^i^	91.03 (13)	C7—C8—H8B	109.5
O3—S1—O2	118.67 (19)	H8A—C8—H8B	109.5
O3—S1—N2	105.18 (18)	C7—C8—H8C	109.5
O2—S1—N2	112.07 (17)	H8A—C8—H8C	109.5
O3—S1—C10	107.86 (19)	H8B—C8—H8C	109.5
O2—S1—C10	106.05 (19)	C11—C10—C15	121.3 (4)
N2—S1—C10	106.37 (18)	C11—C10—S1	119.2 (3)
O4—S2—C17	105.9 (2)	C15—C10—S1	119.5 (3)
O4—S2—C16	102.9 (2)	C10—C11—C12	118.8 (4)
C17—S2—C16	98.1 (2)	C10—C11—H11	120.6
C1—O1—Cu1	121.3 (3)	C12—C11—H11	120.6
S2—O4—Cu1	120.97 (17)	C13—C12—C11	120.3 (4)
C7—N1—N2	117.5 (3)	C13—C12—H12	119.8
C7—N1—Cu1	127.0 (3)	C11—C12—H12	119.8
N2—N1—Cu1	114.7 (2)	C12—C13—C14	120.3 (4)
N1—N2—S1	108.2 (2)	C12—C13—H13	119.9
N1—N2—Cu1^i^	122.8 (2)	C14—C13—H13	119.9
S1—N2—Cu1^i^	105.85 (17)	C15—C14—C13	119.9 (4)
O1—C1—C2	117.5 (4)	C15—C14—H14	120.0
O1—C1—C6	125.2 (4)	C13—C14—H14	120.0
C2—C1—C6	117.3 (4)	C14—C15—C10	119.3 (4)
C3—C2—C1	122.9 (4)	C14—C15—H15	120.4
C3—C2—H2	118.5	C10—C15—H15	120.4
C1—C2—H2	118.5	S2—C16—H16A	109.5
C2—C3—C4	119.0 (4)	S2—C16—H16B	109.5
C2—C3—H3	120.5	H16A—C16—H16B	109.5
C4—C3—H3	120.5	S2—C16—H16C	109.5
C5—C4—C3	120.7 (4)	H16A—C16—H16C	109.5
C5—C4—Br1	119.9 (3)	H16B—C16—H16C	109.5
C3—C4—Br1	119.3 (3)	S2—C17—H17A	109.5
C4—C5—C6	121.5 (4)	S2—C17—H17B	109.5
C4—C5—H5	119.2	H17A—C17—H17B	109.5
C6—C5—H5	119.2	S2—C17—H17C	109.5
C5—C6—C1	118.4 (4)	H17A—C17—H17C	109.5
C5—C6—C7	119.0 (4)	H17B—C17—H17C	109.5
			
N1—Cu1—O1—C1	40.0 (3)	C3—C4—C5—C6	−1.3 (7)
O4—Cu1—O1—C1	−152.5 (3)	Br1—C4—C5—C6	−178.1 (3)
N2^i^—Cu1—O1—C1	−58.2 (5)	C4—C5—C6—C1	−1.1 (6)
C17—S2—O4—Cu1	−58.7 (3)	C4—C5—C6—C7	−177.5 (4)
C16—S2—O4—Cu1	−161.2 (2)	O1—C1—C6—C5	−177.2 (4)
O1—Cu1—O4—S2	−23.1 (2)	C2—C1—C6—C5	3.2 (6)
N1—Cu1—O4—S2	70.5 (7)	O1—C1—C6—C7	−0.9 (6)
N2^i^—Cu1—O4—S2	−176.4 (2)	C2—C1—C6—C7	179.6 (4)
O1—Cu1—N1—C7	−34.4 (4)	N2—N1—C7—C6	−174.7 (3)
O4—Cu1—N1—C7	−128.1 (6)	Cu1—N1—C7—C6	15.4 (5)
N2^i^—Cu1—N1—C7	119.1 (3)	N2—N1—C7—C8	5.6 (6)
O1—Cu1—N1—N2	155.5 (3)	Cu1—N1—C7—C8	−164.3 (4)
O4—Cu1—N1—N2	61.8 (7)	C5—C6—C7—N1	−174.6 (4)
N2^i^—Cu1—N1—N2	−51.0 (3)	C1—C6—C7—N1	9.1 (6)
C7—N1—N2—S1	132.6 (3)	C5—C6—C7—C8	5.1 (6)
Cu1—N1—N2—S1	−56.4 (3)	C1—C6—C7—C8	−171.2 (4)
C7—N1—N2—Cu1^i^	−103.8 (4)	O3—S1—C10—C11	−163.2 (3)
Cu1—N1—N2—Cu1^i^	67.3 (3)	O2—S1—C10—C11	−35.1 (4)
O3—S1—N2—N1	166.0 (2)	N2—S1—C10—C11	84.4 (4)
O2—S1—N2—N1	35.8 (3)	O3—S1—C10—C15	16.4 (4)
C10—S1—N2—N1	−79.7 (3)	O2—S1—C10—C15	144.5 (3)
O3—S1—N2—Cu1^i^	32.7 (2)	N2—S1—C10—C15	−96.0 (3)
O2—S1—N2—Cu1^i^	−97.6 (2)	C15—C10—C11—C12	−0.7 (6)
C10—S1—N2—Cu1^i^	146.95 (18)	S1—C10—C11—C12	178.8 (3)
Cu1—O1—C1—C2	148.7 (3)	C10—C11—C12—C13	−0.9 (7)
Cu1—O1—C1—C6	−30.8 (5)	C11—C12—C13—C14	2.1 (7)
O1—C1—C2—C3	177.1 (4)	C12—C13—C14—C15	−1.7 (7)
C6—C1—C2—C3	−3.3 (6)	C13—C14—C15—C10	0.1 (7)
C1—C2—C3—C4	1.1 (7)	C11—C10—C15—C14	1.1 (6)
C2—C3—C4—C5	1.3 (7)	S1—C10—C15—C14	−178.5 (3)
C2—C3—C4—Br1	178.2 (3)		

Symmetry codes: (i) −*x*+2, −*y*+1, −*z*.
